# Introducing and Expanding Newborn Screening in the MENA Region

**DOI:** 10.3390/ijns6010012

**Published:** 2020-02-19

**Authors:** Victor Skrinska, Issam Khneisser, Peter Schielen, Gerard Loeber

**Affiliations:** 1 Metabolic Laboratory, Department of Pathology and Laboratory Medicine, Hamad Medical Corporation, Doha P.O. Box 3050, Qatar; vaskrinska@gmail.com; 2 Newborn Screening Laboratory, Medical Genetic Unit, Faculty of Medicine, Saint Joseph University, B.P. Riad El Solh, Beirut 11-5076, Lebanon; issam.khneisser@usj.edu.lb; 3 Head Reference Laboratory for Neonatal Screening, Center for Health Protection, National Institute for Public Health and the Environment (RIVM), P.O. Box 1, 3720 BA Bilthoven, The Netherlands; peter.schielen@rivm.nl; 4 ISNS Office, 3721 CK Bilthoven, The Netherlands

This special edition of the *International Journal of Neonatal Screening* includes the presentations of the fourth Meeting of the Middle East North Africa (MENA) Region of the International Society for Neonatal Screening (ISNS) held in Limassol, Cyprus, March 8–11, 2020. After the first three MENA meetings in Marrakech (2006), Cairo (2008), and Doha (2010), organised by and with substantial financial support from the US National Institute for Child Health and Human Development, due to political unrest it took ten years to find the necessary finances and a suitable location for this fourth MENA meeting.

The prevalence of treatable genetic disorders in the Region is high, in part caused by a relatively high degree of consanguinity. There is wide variability in neonatal screening programmes, which range from highly advanced to very limited.

This meeting provides a common forum for all of the regional screening programmes to find information and resources, and for an open exchange of experience and expertise that benefits programmes at an early stage of development, as well as those that are already at an advanced stage. The format includes key lectures, oral and poster presentations, and workshops. This exchange seeks to benefit the children of the region by introducing, expanding, and improving neonatal screening.

The abstracts, grouped as oral presentations and poster presentations, are included below.

## Oral Presentations

### O1. History of Newborn Screening: Newborn Screening and Public Health Infrastructure Initiatives

BonhamJ. R.Sheffield Children’s Hospital, Sheffield, United Kingdom

The courage and commitment of parents wanting the best possible chance for their children has helped begin and develop newborn screening programs across the world since PKU was first recognised by Asbjorn Folling in 1934 and continues to promote the use of modern developments such as genetic technology to identify and characterise rare and life threatening diseases. 

The range of conditions that can be considered as candidates for screening continues to grow as new treatments and technological advances in detection provide effective ways to intervene while patients remain asymptomatic, thus avoiding the serious consequences often seen when patients are recognised following a clinical presentation. Significant developments such as automated immunoassay and the introduction of electrospray ionisation linked to tandem mass spectrometry have greatly extended the potential to undertake this screening on a whole population basis for all newborns at an affordable cost.

Nevertheless, early intervention in apparently healthy newborns can plunge families into unfamiliar and frightening territory and only by the meticulous management of carefully constructed public health programs with explicit key performance indicators can quality be assured and significant harm avoided.

In this presentation we will look at some of the benefits and challenges posed both by well-established technologies and newer techniques such as next generation sequencing when implementing new programmes including the need to be clear about case definitions from the outset, the need to plan long term outcome studies to assess and improve the effectiveness of screening and the importance of considering communication from a parents’ perspective.

### O2. Newborn Screening in Cyprus

ArgyriouArgyrisCentre for Preventive Paediatrics, Limassol, Cyprus

The Cypriot neonatal metabolic screening program started in Cyprus in 1988 as a civil society effort by the Centre for Preventive Paediatrics, a charity. The program screens for Congenital Hypothyroidism (CH) and Phenylketonuria (PH). The Centre is responsible for inviting parents to participate in the screening process, in identifying screen positive newborns, diagnosing the condition, communicating with the physicians, assess long term treatment efficacy and educate the public regarding the usefulness of screening programs as well as participating in forming public health policy. We will present the results of screening more than 281,000 neonates over a period between from 1988 to 2019. During this period we detected 152 cases of CH of which 123 cases have been confirmed by clinical and imaging studies. During the same period we detected 3 cases of classic PH, 5 mild PH and 11 hyperphenylalaninemia cases. We will present the performance of the screening as well as the results of the clinical and follow up care and the future plan to expand the panel of testing to include a further set of conditions.

### O3. Selective Screening for Inborn Errors of Metabolism in Cyprus

DrousiotouAnthiDepartment of Biochemical Genetics, The Cyprus Institute of Neurology & Genetics, Nicosia, Cyprus

Systematic screening for inborn errors of metabolism (IEM) began in Cyprus in 1990, with the establishment of the first laboratory of Biochemical Genetics at the Cyprus Institute of Neurology & Genetics. Through the introduction of the basic diagnostic tests and collaboration with laboratories abroad for the more specialized tests, it became possible to offer full investigation for patients suspected of having an IEM. Over the last thirty years, about 5000 patients were investigated and 150 diagnoses were made (diagnostic yield about 3%). If we exclude the hyperphenylalaninaemias that were diagnosed following a positive newborn screening result, the most common groups were the organic acidurias (16%) and the lysosomal storage disorders (16%), followed by the carbohydrate disorders (13%) and the mitochondrial disorders (10%). Among the aminoacidopathies, the most common were the hyperphenylalaninaemias (55%) followed by maple syrup urine disease (15%). Among the organic acidurias, glutaric aciduria type I was the most common (42%) followed by methylmalonic aciduria (21%). Some disorders were found to have a relatively high incidence in specific communities, for example Sandhoff disease among the Cypriot Maronites and GM1 gangliosidosis in one particular area. Other disorders were found to have a relatively higher overall incidence, compared to other Caucasian populations, for example glutaric aciduria type I and galactosaemia, while others were found to have a relatively lower incidence, for example Gaucher disease. Molecular characterization of selected disorders revealed many novel mutations, specific to the Cypriot population. The existence of a local reference laboratory for the investigation of IEM has had a positive impact on the diagnosis, monitoring and prevention of IEM in Cyprus.

### O4. The Status of Newborn Screening Programmes in the MENA Region, Country Reports

#### a. The Status of Newborn Screening Program in Iran

KobarfardFarzadMetabolic Disorders Screening Laboratory, Shahid Beheshti University of Medical Sciences (SBMU), Tehran, Iran

Newborn screening for inherited metabolic diseases (IMDs) started in Iran covering CH and PKU in 2005. However, screening for expanded panels of IMDs, using tandem mass spectrometry was set as the agenda for the Iran Ministry of Health (MOH) in 2017. The required infrastructures were set in two directions: laboratory screening and clinical screening.

Ten labs were qualified for nationwide screening of the expanded panel after strict assessment by MOH technical authorities. Each laboratory was linked to a referral hospital and a panel of metabolic specialists in the hospital. Local health centers were trained for sample collection and transferring the samples to the laboratories. The screening program was launched as a pilot study in 6 provinces of Iran in 2017 and a total of 145,169 babies were screened for the expanded panel of IMDs in two years.

Positive samples were 3379 (2.3%) and 82 cases were confirmed as metabolic diseases (1 in 1770). The prevalence of IMDs were as follow: 1 for IVA, 22 for HPA/PKU, 7 for GA1, 2 for CUD, 2 for NKH, 12 for MSUD, 1 for GA2, 3 for PA, 6 for MMA, 4 for TYR, 4 for MCAD, 6 for UCD.

Multilateral efforts are being made to identify and overcome the possible obstacles and challenges for implementation of nationwide screening in MOH at administrative and executive levels, as well as the screening laboratories and hospitals. 

#### b. Newborn Screening in Lebanon: 25 Years Summary

KhneisserIssamNewborn Screening Laboratory, Medical Genetic Unit, Saint Joseph University

In 1995, Newborn Screening in Lebanon started covering CH, PKU, G6PD and GAL (1997) for a symbolic fee paid by families’ out-of-pocket money. The expansion to tandem mass spectrometry took place in 2007 (with no extra fees). In Late 2018, a National Screening Program for SCID had been launched with the Ministry of health and AUBMC and USJ, while AUST do only the metabolic screening. Currently, 60 per cent of Lebanese babies born in Lebanon are covered by these programs.

Since 1995, USJ Screened 408,000 newborns. 5021 G6PD, 219 CH, 43 PKU and 7 GAL cases had been identified. Among the 276000 babies screened for the expanded MSMS panel, 19 MSUD, 6 CIT, 3 to 5 cases for each disease (MCAD, VLCAD, IVA, MMA, PA, GAI and HMG), 2 BKT, one CPT-I and one LCHAD case were found. The PPV of MSMS is remarkable, at 0.7 in favor of this voluntarily program. The incidence of MCAD was lower than expected, and relatively high consanguinity was found in a cluster of 18 of 19 MSUD cases that had been identified in a geographically isolated area of 40,000 habitants.

Continuous efforts are undergoing to increase coverage facing many economical, logistics and health policy challenges.

#### c. Newborn Screening in Qatar, 2020 Update

MitriRolaMetabolic Laboratory, Department of Laboratory Medicine and Pathology, Hamad Medical Corporation, Doha, Qatar

The Metabolic Laboratory at Hamad Medical Corporation serves as the National Newborn Screening Laboratory for the State of Qatar. Approximately 28,000 newborns per year are screened in Qatar. Screening is provided for all newborns of residents at no cost. Typically more than 98% of the births are screened. The current screening panel of 83 disorders performed by the Laboratory includes Galactosemia, Biotinidase Deficiency, Congenital Hypothyroidism, Congenital Adrenal Hyperplasia, 25 Amino Acid Disorders, 16 Fatty Acid Oxidation Disorders, 25 Organic Acidemias, and 12 Hemoglobinopathies. Guanidinoacetate Methyltransferase Deficiency, Severe Combined Immunodeficiency, and 4 Lysosomal Storage Diseases are currently under validation. The screens are performed using a wide range of technologies including photometric techniques, liquid chromatography tandem mass spectrometry, and liquid chromatography. 2nd tier testing that provides methylmalonic acid and methylcitric acid levels are performed as a reflex for suspicious screens with abnormal levels of methionine, homocysteine, or propionylcarnitine to differentiate a disorder and to reduce false positives. The Laboratory is currently evaluating and validating a reflex to a rapid targeted next generation sequencing to improve performance metrics.

#### d. Newborn Screening in United Arab Emirates

SalaheldinMohamedNewborn Screening &Genetic Center, Purehealth, MOHAP, United Arab Emirates

The National Neonatal Screening Program in United Arab Emirates was launched in January 1995 and covered the following diseases at the national level: Phenyl ketonuria (1995), Congenital hypothyroidism (1998), Sickle cell disease (2002), Congenital Adrenal Hyperplasia (2005), Biotinidase Deficiency (2010), Tandem mass spectrometry (MS/MS) for AA, FA&OA (2011), Galactosemia (2016) & G6PD deficiency (2019). Plus Cystic Fibrosis as pilot study in 2020.

Currently, every newborn is screened for more than 40 diseases. This is in addition to congenital heart disease and hearing screening programs.

The protocol includes systematic outcome evaluation of all phases (Pre-analytical, analytical and post-analytical). The quality assessment of the National Neonatal Screening Laboratory is under the CDC evaluation.

Since 1995, more than 2700 infants were saved from mortality and/or physical, mental and other associated morbidities through screening of more than 1,300,000 infants with 86% uptake in 2019.

The following diseases have been identified: 653 Congenital Hypothyroidism cases (1:2075), 101 Congenital Adrenal Hyperplasia (1:8743), 75 Biotinidase Deficiency ((1:8142), 284 Amino Acid, Organic Acid and Fatty Acid cases (MS/MS) (1:2377), 86 Phenylketonuria (PKU) (1:14,523), 429 sickle cell diseases (0.44/1000), 9319 sickle cell traits (7.8/1000), 77 β–thalassemia diseases, 12 Galactosemia (1:12,925) and 1145 Glucose-6-Phosphate Dehydrogenase Deficiency cases.

### O5. Experiences, Hopes, Despair and Challenges to Implement Newborn Screening Programs in the Countries of the MENA Region

ChabraouiLayachiUniversity Hospital of Rabat, University Mohammed V Rabat, Morocco

The objective of neonatal screening is to identify infants with medical conditions in order to ensure early treatment. Each country must organize its program according to its priorities, taking into account the prevalence of diseases and the cost benefit ratio. A national neonatal screening (NBS) program requires perfect organization and consistent funding. In developing countries like most of those in the MENA region, funding remains an obstacle to setting up a real neonatal screening program.

In Morocco, when we became interested in NBS (since 1990), we realized that the diseases to be screened were unknown to all of the potential stakeholders. We then started by making paediatricians aware of the need to diagnose and take care of patients with inherited metabolic diseases. We supervised several thesis works on divers IEM subjects and we organized several educational scientific meetings. We progressively presented the results of our work at national and international congresses. We have also invited representatives of the health authorities to each event that we have organized. We have emphasized the high incidence of congenital hypothyroidism and phenylketonuria and the need to systematically screen for these two diseases.

The Ministry of Health has finally drawn up a plan of action/protocols after the 2006 Marrakech conference. They set up a national coordination committee which worked in collaboration with the JICA, so a regional hypothyroidism screening program started in 2012 in the Rabat-Salé-Zemour-Zaïr region. The program was gradually and not without difficulties, extended to three other regions of the country.

### O6. The Impact of Consanguinity on the Incidence of Inherited Metabolic Disorders: Challenges and Opportunities

Ben-OmranTawfegDivision Chief, Genetic and Genomic Medicine, Sidra Medicine and Hamad Medical Corporation, Doha-Qatar

Consanguinity and endogamy are high in the Middle East-North Africa (MENA) region including the Gulf countries and range between 20% and 60%. In many MENA countries, consanguineous marriages are culturally favored with longstanding traditions, for example, in Qatar the rate of consanguinity is relatively high with a rate of 54%, and predominantly first cousin marriages comprising 26.7% of all marriages. Together with consanguinity, the relatively large family size plays a role in the high prevalence of autosomal recessive conditions in the population of the region. Additionally, the high rate of consanguinity favors the coexistence of multiple distinct autosomal recessive disorders within single families. Even more complex families have been diagnosed with a combination of autosomal recessive disorders and other single gene disorders with different inheritance patterns. The recent advances in genomic sequencing technologies have helped identify patients affected by more than one genetic condition. On the other hand, many countries in the MENA region have served as key areas for the discovery of novel genetic causes of human disease. In many cases, these causes were first identified in this region through clinically oriented research endeavors and then extrapolated to other parts of the world. That is, after identifying individuals with causative genetic changes in the population of the region, individuals in other parts of the world with the same phenotypes were found, tested, and identified to have the same underlying genes involved, though sometimes with compound heterozygosity rather than homozygosity for pathogenic variants. It is imperative to advocate for a change to the cultural and social framework that reproduces and normalizes consanguineous marriages in the region. Policy makers should endorse social, educational, and public health initiatives to mitigate the impact of genetic disease in the MENA region, as well as establishing preventive programs like premarital and newborn screening.

### O7. Congenital Hyperthyroidism (CH) in Iran

KobarfardFarzadMetabolic Disorders Screening Laboratory, Shahid Beheshti University of Medical sciences (SBMU), Tehran, Iran

Congenital Hypothyroidism (CH) is the most prevalent endocrine disease and the leading cause of preventable mental retardation. With the introduction of CH neonatal screening programs, babies affected with this condition are detected before the clinical manifestations are evident and irreversible.

The national newborn screening program for CH in Iran started in 2005 after two years of planning and pilot study. The coverage of the program is currently 97.7% of all the newborns in Iran and the prevalence of CH (both transient and permanent) is 2.9 in 1000. 

85% of the samples are collected during the first 3–5 days of birth and in 78.5% of the positive cases, treatment has been started before 28th day after birth. In total ca 15 million newborns have been screened since the program started and ca 45,000 positive case have been diagnosed and the appropriate actions have been taken for their treatment. One third of the positive cases have been determined as transient congenital hypothyroidism.

### O8. Neonatal Screening for Congenital Hypothyroidism: Experience from Morocco

TantaneAsmae^1^DghoughiNouzha^2^BeneradiRajae^1^CherradiNajat^1^MessaoudiFatima Ezzahra^1^RhajaouiMohamed^1^1National Institute of Hygiene of Morocco2Direction of Population of Morocco

Neonatal screening aims to systematically search all neonates of the general population for a congenital pathology of early revelation, before it causes irreversible sequel. It is a prevention and public health action to identify and treat birth defects due to certain diseases. In Morocco, in 2006 and following the organization of the First Conference on Enhancing Neonatal Screening in North Africa and the Middle East, the Ministry of Health decided that it would be very relevant to introduce systematization of tests for congenital anomalies, which lead to the introduction of neonatal congenital hypothyroidism screening according to the 2012–2016 action plan: Measure 12 of Axis 3 concerning the reinforcement of neonatal surveillance during the postpartum period, to accelerate the reduction of neonatal mortality.

Congenital hypothyroidism is a hypofunction of the thyroid gland marked by insufficient production of thyroid hormones. Screening is done by the determination of TSH systematically in births in five regions of Morocco by a capillary blood sample at the heel. For example, at the Rabat Salé Kenitra region, twenty-one newborns have been able to escape mental handicap thanks to this screening, the management of which is still at the parents’ expense.

The Ministry of Health has taken an important step in mastering the basic principles and organization of the neonatal screening process, but it is necessary to strengthen some of organizational, communication and monitoring aspects.

### O9. Determining Reference Ranges for tT4 in Dried Blood Samples of Newborn Screenings

FingerhutRalphHijmanAnna-IsabellaKonradand DanielUniversity Childrens Hospital, Swiss Newborn Screening Laboratory, Zurich, Switzerland

The purpose of this study was to define reference values for total thyroxine (tT4) in dried blood samples (DBS) taken for newborn screening. The Background for our research question was the possible benefit to measuring tT4 concentrations for premature and term born infants directly from dried blood samples taken for newborn screening. In order to have a sufficient number of samples for the extremely premature infants (<30 weeks) we set up a retrospective study, measuring the concentrations of DBS taken over the previous 21 weeks. This time frame was a result of the included miniature study of tT4 stability in DBS. Our research showed that tT4 strongly correlates with gestational age in premature infants, highlighting the need for age specific reference ranges. For term born infants the tT4 ranges did not vary significantly for different gestational ages, allowing one single reference range.

### O10. Congenital Adrenal Hyperplasia Screening

TorresaniToniHinteregg, Switzerland

Congenital adrenal hyperplasia (CAH) is a group of recessively inherited disorders of adrenocortical steroidogenesis caused by various enzyme deficiencies, mainly by 21-hydroxylase deficiency (21-OHD). The worldwide incidence of classical congenital adrenal hyperplasia (C-CAH) is estimated to be between 1:10.000 and 1:16.000 live births. Early diagnosis and treatment are necessary to prevent adrenal crisis and early infant death in the classical salt wasting form of CAH.

Newborn screening (NBS) for CAH has been available internationally for over 30 years, and is currently implemented in Europe, New Zealand, the USA and in many countries in Asia and Latin America. Supporting NBS is the observed difference in incidence, sex ratio and disease spectrum in screened versus unscreened populations.

Newborn screening for CAH is usually done by means of 17-hydroxy-progesterone (17-OHP) determination in dried blood on filter paper. Methodological problems of false-positive samples, especially in pre-term infants, can be corrected by adapting the cut-off values for 17-OHP to birth weight, gestational age and age at the time of collection and optionally by performing a second-tier screening.

### O11. Cystic Fibrosis Screening

FingerhutRalphSwiss Newborn Screening Laboratory, and Children’s Research Center, University Children’s Hospital, Zurich, Switzerland

Cystic fibrosis (CF) is one of the most common autosomal recessive disorders with a frequency of about 1 in 3500 livebirths in the white population. Switzerland started CF-NBS in January 2011. Due to the low specificity of IRT as a marker for CF, there are several possible workflows for CF screening: IRT–IRT; IRT–IRT–DNA, IRT–DNA; IRT–PAP–DNA. Switzerland has chosen the IRT – DNA protocol with subsequent direct referral to sweat testing, with an additional fail safe step. From 2011–2019 in total 784,328 newborns have been tested in Switzerland, and 216 cases with CF have been confirmed, which gives an incidence of 1:3613.

Still there is one problem with determination of IRT from dried blood spots. We observed a high variation in IRT determination, which is also reflected by high variation of results from different external quality control schemes with different commercial test kits.

### O12. Neonatal Screening for Severe Combined Immunodeficiency: Challenges in the Setting of a Country with Low Resources

DbaiboGhassanReslanLinaThe National Neonatal Screening Program for Primary Immunodeficiency Disorders (NaSPID) in Lebanon

Severe Combined Immunodeficiency Disease (SCID) is the most severe form of Primary Immunodeficiency Diseases (PID), characterized by absent/dysfunctional T cells and/or B and NK cells. Newborns with SCID present with failure to thrive and severe, often fatal infections. Early diagnosis and treatment are therefore essential. 

Screening for SCID is of value particularly in the Middle East and North Africa (MENA) region, where a relatively high frequency of consanguineous marriages exists. In 2018, Lebanon achieved a milestone in becoming the first country in the region to establish the **Na**tional Newborn **S**creening **P**rogram for Primary **I**mmunodeficiency **D**iseases (NaSPID) partly supported by the Lebanese Ministry of Public Health to screen for SCID based on the quantification of T-cell receptor excision circles (TRECs), biomarkers for newly developed T-lymphocytes.

Since the program launching, more than 15,000 newborns were screened at the two designated laboratories. So far, the screening has identified one case of confirmed DiGeorge Syndrome in addition to two suspected SCID cases that need confirmatory testing.

NASPID is still in its infancy. Significant challenges were faced in the optimal implementation. These include logistical challenges, acceptance by families and physicians, as well as financial challenges. These challenges will be reviewed. 

### O13. Newborn Screening for SCID in the Polish-German Trans-Border Area: Experiences from the First Year of Collaboration

GiżewskaMariaDurdaKatarzynaWinterTheresaOstrowskaIwonaOłtarzewskiMariuszKleinJeannetteBlankenstainOliverRomanowskaHannaKrzywińska-ZdebElżbietaPatalanMichałBartkowiakElżbietaTrafnyNataliaSeiberlingStefanDomańskiGrzegorzBirkenfeldBożenaNauckMatthiasBernuthHorst vonMeiselChristianBernatowskaEwaPacMałgorzataWalczakMieczysławPomeranian Medical University in Szczecin, Poland, Department of Paediatrics, Endocrinology, Diabetology, Metabolic Diseases and Ca, Szczecin, Poland

In 2017, in the Polish-German trans-border area of Mecklenburg-Vorpommern, Brandenburg and West Pomerania, in collaboration with 2 centers in Warsaw, a partnership in the field of newborn screening (NBS) for severe combined immunodeficiency (SCID) was started. It was implemented based on the EU founded Interreg Va project “Innovative Polish-German cross-border program for early diagnosis and treatment of rare diseases in newborns–RareScreen” (INT 10) which involves expanding the NBS panel for inborn errors of metabolism and other rare diseases including SCID for the children in the founding area.

The SCID NBS test is based on real-time polymerase chain reaction (qPCR) and simultaneous measurement of T cell receptor excision circles (TREC), kappa-deleting recombination excision circles (KREC) and beta-actin (ACTB) as a quality marker of DNA. DNA is extracted from blood spots collected on NBS filter paper. This method is effective in NBS for SCID patients and other primary immunodeficiency disorders as Nijmegen-breakage-syndrome, X-linked agammaglobulinemia (XLA) and ataxia-telangiectasia.

During the first year of the ‘RareScreen’ project, 39.899 newborns were screened for SCID. Sixty-one re-calls included 38 newborns with TREC and/or KREC values below the cut-off and 23 due to poor quality of samples. After retesting, 9 newborns were referred to further evaluation. Confirmatory procedures proofed diagnosis of SCID (T-B-NK +) with severe rhizomelic skeletal dysplasia and Nijmegen-breakage-syndrome. In 3 children final diagnosis including molecular tests is pending. Two positive results were related to prematurity (Hbd 26) and mother immunosuppression during pregnancy, 2 had normal control results. The overall positive predictive value (PPV) was 7.89%. This is the first screening study allowing identification of newborns with SCID in a large region of central Europe as well as giving valuable knowledge of its feasibility.

### O14. Aminoacidopathies, Fatty Acids and Organic Acid Oxidation Disorders Screening Using MS/MS, Updates and New Developments

KhneisserIssamNewborn Screening Laboratory, Medical Genetic Unit, Saint Joseph University.

Tandem mass spectrometry applications are increasing the number of newborn screened diseases. During the last two decades, application shifted from derivatized method only, to more widely used non derivatized method, using less harmful solutions for human and less corrosive for the equipment. This technology permits to detect the substrate and its product, the use of ratios improves the PPV and reduces the interference of endogenous and exogenous biological factors. New possibilities of disease detection had been added lately, reaching 19-plex for LSD and MPS, in addition to other peroxisomal, carnitine and purine metabolism defects. Second tier analysis is commonly used to differentiate between isobaric molecules and improve diagnosis. Recently, some MSMS equipment may run first and second tier in the same run and this will be in favor of the timely starting treatment as soon as possible.

More pharmaceutical findings of new treatment will lead to more candidate diseases to be added to newborn screening by MSMS.

### O15. Phenylketonuria Screening in the Republic of Kazakhstan

MurtazaliyevaAlexandraSvyatovaGulnaraKirikbayevaMeruertScientific Center of Obstetrics, Gynecology and Perinatology, Republican Medical Genetic Consultation, Almaty, Kazakhstan

Introduction: Phenylketonuria (PKU) is an autosomal recessive inborn error of metabolism resulting from a deficiency of phenylalanine hydroxylase (OMIM), impairs postnatal cognitive development, which can be prevented by early and continuous treatment. Therefore mass newborn screening for phenylketonuria has been introduced in Kazakhstan since 2007.

Methods: Mass neonatal screening for PKU is performed by immunofluorescence method with standard kits (Neonatal Phenylalanine kit, PerkinElmer) on DBS in every region of Kazakhstan.

Results. In 2017–2019 nearly 1 million newborns were studied for PKU. Since 2017 the average coverage of neonatal screening in Kazakhstan increased from 87% to 92.5%. Kazakhstan has 16 regions and 3 megalopolis. The coverage of PKU in these regions is different, but only in 3 regions the average coverage is less than 90%. The effective state program of neonatal screening allowed to decrease treatment initiation until <21 days of life. The average rate of PKU in Kazakhstan is 1:24,000 newborns. The rate in regions varies significantly from 1:65,310 in Nur-Sultan to 1:5020 in Kostanay regions. This variability depends on ethnic populations structure in different regions of Kazakhstan. All patients have been treated with low phenylalanine diet, 1 patient receives patogenic treatment with sapropterin dihydrochloride in dosages of 10 mg per kg. The results of this study confirm the benefit of early detection and treatment of PKU through the screening program. All costs associated with neonatal screening for PKU and patient care are covered by government assistance.

Discussion. Monitoring treatment patients with PKU using Tandem Mass Spectrometry method will allow more accurate controlling the diet therapy and prevent metabolic disorders. Ethnic difference in Kazakhstan’s population are important for molecular genetic diagnostics.

### O16. Expansion of the Dutch Newborn Screening Panel: The First Months of Screening for CPT-I, PA, MMA

MaaseRoseBouvaMarelleHodemaekersHennieVeldenMonique de Sain-van derSpronsenFrancjan vanVerschoof-PuiteRendelienGorpAnkie vanSchielenPeterDutch National Institute for Public Health and the Environment (RIVM), Department Biologicals, Screening and Innovation (BSI) | Centre for Health Prote, Bilthoven, Netherlands

In the MENA region, the consequences of and possibility to treat of Carnitine palmitoyltransferase I deficiency (CPT-I), Propionyl-CoA carboxylase deficiency (PA) and Methylmalonyl-CoA mutase deficiency (MMA) warrant their inclusion in a number of newborn screening (NBS) programs. In 2019 these 3 conditions, were added to the Dutch NBS panel. Screening for CPT-I, PA and MMA is based on measurement of acylcarnitine concentrations and acylcarnitine ratios in heel prick blood, using a pre-existing analytical technique, tandem mass spectrometry (MS/MS; Waters Xevo TQD, PerkinElmer Neobase2 non-derivatized kit). A pre-implementation study using C3, C3/C2 and C3/C16 as first tier for PA and MMA NBS, showed a high number of false positive referrals. To diminish the high number of false positives, the PA and MMA NBS algorithm was based on first tier test, conducted at all five regional Dutch NBS laboratories, followed by a second tier test to measure methylmalonic acid (MMAMB) and 2-methylcitric acid (MCA) (in house LC MS/MS method, Dutch NBS Reference Laboratory only). Method: Test performance of both first and second tier tests are reviewed for the first 5 months of national NBS for CPT-I, PA and MMA. Each referral for CPT-I, PA and MMA was reviewed. Results: The establishment of national NBS for CPT-I, PA and MMA, including logistical considerations related to MMAMB/MCA screening at a single site is presented. In the first 6 weeks of NBS there were 4 referrals (3 PA, 1 MMA); 1 was true positive (PA) and 3 were false positive referrals. The results from the first 5 months of screening (approximately 70,000 screens) will be evaluated, including performance of the screening algorithm, referrals and potential required amendments to the algorithm, based on short-term follow-up of referrals. Conclusions: Based on the pre-implementation study, less than 10 referrals had been expected for CPT-I, PA and MMA annually. Data from the first 5 months of NBS for CPT-I, PA and MMA are relevant to better understand the suitability of the NBS algorithms.

### O17. Lysosomal Storage Disease Screening, Update and Latest Developments

MitriRolaMetabolic Laboratory, Department of Laboratory Medicine and Pathology, Hamad Medical Corporation, Doha, Qatar

Screening for lysosomal storage diseases (LSD) is rapidly expanding worldwide due to advances in screening technology and treatment options that lead to better clinical outcomes when initiated shortly after birth. Screening for LSD using dried blood spots (DBS) is typically performed by measuring enzyme activity through incubation of DBS extracts with synthetic substrates and detecting the products by mass spectrometry or digital microfluidic photometry. The Metabolic Laboratory in Qatar is currently validating a digital microfluidic system for screening for Pompe, Fabry, Gaucher, and Mucopolysaccharidosis Type I.False positive rates associated with LSD screening are a significant concern. In addition, the occurrence of pseudodeficiencies, which are positive screens due to reduced enzyme activity with synthetic substrates but have normal activity with endogenous substrates, contribute to false positive rates. To minimize false positive rates as well as confirm suspected positive screens, the Metabolic Laboratory has established a screening protocol that incorporates targeted next generation sequencing (tNGS) as a reflex confirmatory test for all suspected positive screens for LSD. The reduced cost and rapid resulting of tNGS allows optimization of LSD screening on the initial DBS.

### O18. Targeted-Population Screening for Mucopolysaccharidoses—Paving the Way to Newborn Screening

LukacsZoltanCobosPaulina NievesSanterRenéOlivaPetraKasperDavidHamburg University Medical Center, Newborn Screening and Metabolic Diagnostics, Hamburg, Germany

Lysosomal storage diseases present with highly variable symptoms and usually with different severity, depending on the individual age of onset. Diagnosis is sometimes further delayed because of the relative rarity of the diseases which does not make them prime candidates in diagnostic algorithms. Recently, dried blood specimens have been introduced for multiplexed enzyme activity measurement of a number of lysosomal enzymes and thereby, help to facilitate testing for MPS. Dried blood cards have been shipped from distant health care providers (Middle East, Russia, Southern Europe, South Africa, among others) to the metabolic laboratory at the Hamburg University Medical Center. Patients showed symptoms compatible with mucopolysaccharidoses (MPS), esp. coarse face, hepatosplenomegaly, and rheuma-like symptoms without inflammation. The samples were tested for MPS I, MPS II (male) and MPS VI using fluorometry or tandem mass spectrometry. Among 9832 samples tested to date, 6.9% have tested positive for MPS I, 3.2% tested positive for MPS II, 1.1% for MPS IVA, 4.6% for MPS VI and 2.3% showed enzyme activities compatible with mucolipidoses II/III. In addition, 18 samples were suspicious for multiple sulfatase deficiency. As beta-galactosidase is run as a reference enzyme, several cases of potential GM1-gangliosidosis/MPS IVB have been detected. Targeted-population screening of patients with symptoms remotely associated with the distinct lysosomal storage diseases using DBS proved a highly efficient approach in expediting the diagnosis of patients. In addition, it is cost effective, and can provide a potential alternative to newborn screening, when this is locally not an option for financial or ethical reasons. Furthermore, it paves the way to newborn screening by establishing technology, knowledge in the laboratory and raising awareness among clinicians.

### O19. Screening and Diagnosis of Lysosomal Storage Disorders Using a Two-Tier Screening Strategy of Enzyme Activity & Metabolites by Tandem Mass Spectrometry (MS/MS)

RanieriEnzoMasEmilieSA Pathology Women’s & Children’s Hospital, Biochemical Genetics, Genetics & Molecular Pathology, Adelaide, Australia

Lysosomal storage disorders (LSD’s) are a group of over 50 disorders caused by the specific deficiency of enzymes (& co-factors) within the lysosome. The estimated incidence is 1 in 7000 in the Australian population, even higher in certain ethic groups.

The use of MS/MS enables the determination of more than 12 LSD enzyme activities performed in two separate multiplex assays from DBS. In the first multiplex assay 6 LSD enzymes (Perkinelmer 6-plex LSD reagents) are galactocerebroside b-galactosidase (Krabbe disease), acid α -galactosidase A (Fabry disease), acid sphingomyelinase (Niemann Pick A/B disease), acid α-glucosidase (Pompe disease), α -L-iduronidase (mucopolysaccharidosis type I) and acid b-glucocerebrosidase (Gaucher disease). In the second, a further 6 LSD of the mucopolysaccharidosis for MPS II, IIIB, IVA, VI, VII and TPP1 (CLN2). This study used de-identified newborn DBS from an unaffected population and from confirmed positive LSD cases that included GALC (5), GLA (14) & carriers (28), ASM A/B (1), IDUA (6), GAA (20) and ABG (20), MPS II (3), MPSIIIB (2), MPS VIA (1), MPSVI (2) and TPP1 (1). All 12 LSD analytical assays gave performance within CV% of 12–15%, with greater than 3 orders of magnitude in dynamic range. The unaffected normal population gave the 1st centile in IU/hr/L whole blood for each of the enzymes as; ABG 2.2, IDUA 1.3, GAA 4.1, ASM 0.8, GALC 1.8 & GLA 1.3, reference ranges was also determined for MPS II, IIIB, IVA, VI, VII and TPP1. The majority of the LSD cases had activities below the 1st centile of the respective unaffected population. A limitation of the 1st tier enzyme assay is the identification of pseudo-deficiencies and late onset disease. The use of 2nd tier MS/MS metabolite determinations of the glycosaminoglycans (mono-disaccharides of heparin, dermatin/chondroitin) and glycosphingolipids (lysoGb3, spinhgomyelin 508, Lyso-PC and sulphatides) can be used on DBS, plasma and urine resulting in the reduction of false positives and provided the confirmatory diagnostic testing for these LSD.

### O20. Advances in Treatment of Genetic Disorders: Lessons Learned from Neuromuscular and Lysosomal Disorders

Ben-OmranTawfegDivision Chief, Genetic and Genomic Medicine, Sidra Medicine and Hamad Medical Corporation, Doha-Qatar

Advances in treatment for neuromuscular and lysosomal disorders hold promise for children with these disorders, so early and accurate genetic diagnosis will allow these treatments to be most effective. Spinal muscular atrophy (SMA) is a devastating autosomal recessive disease that can lead to disability and death, particularly in its most severe form. However, the availability of new treatment like antisense oligonucleotide and gene therapy, both are changing the natural history of the disease and proven to be most effective if given early to presymptomatic patients. Thus, and to avoid the known diagnostic delay and provide optimal effectiveness of these treatments, early identification of patients through NBS will be necessary. Similarly, enzyme replacement therapy (ERT) is at present available therapy for several lysosomal storage disorders. It is more effective if it is started early, in a pre-symptomatic phase which is mainly achieved by newborn screening. In this talk, we will present our experience in treatment of SMA and LSDs in Qatar and highlight the importance of establishing this in presymptomatic infants through establishing newborn screening.

### O21. Haemoglobinopathies Screening in MENA Region

SalaheldinMohamedNewborn Screening & Genetic Center, Purehealth, MOHAP, United Arab Emirates

Patients with sickle cell disease and non-sickling disorders, as well as carriers of abnormal hemoglobin variants, can be detected by NBS. Choosing primary and secondary target diseases influences false negative and false positive results of the overall screening program and, therefore, are important to the evaluation of the screening process.

Hemoglobinopathies, specifically HbSS, HbS/β-thalassemia and HbSC disease were added to the Recommended Uniform Screening Panel (RUSP) in 2006. Additional hemoglobinopathies readily detected by newborn screening were also added as secondary targets. Examples include HbE disease, HbC disease, HbSE disease, etc.

Sickle cell anemia is common in the MENA World’s population with rates of sickle cell trait ranging from 0.3 to 30%. This high frequency could be related to the high consanguinity rates (20–50% of all marriages).

NBS programs for sickle cell anaemia and trait have been implemented in a number of MENA countries allowing both the prophylactic management of diseased infants and counselling for carrier parents.

National Hb NBS programs with extensive screening coverage are now present in Bahrain, Kuwait, Qatar, Saudi Arabia and the United Arab Emirates. Pilot Hb screening projects have been completed in Jordan, Lebanon, Tunisia and Oman. Little is known about Hb screening activities in other countries.

Newborn screening service for sickle cell disease is still patchy and inadequate in many MENA countries recommending the upgrade of this services with strengthening of the education and training of health care providers and raising public awareness on the feasibility of prevention and care for haemoglobinopathies.

### O22. Haemoglobinopathies—Screening Methodology

MoatStuart JUniversity Hospital of Wales, Cardiff, Wales, CF14 4XW, UK

The aim of newborn screening (NBS) for haemoglobinopathies is to identify infants with the disease states to ensure early treatment. Methods such as HPLC and isoelectric focusing rely on the separation of intact haemoglobin (Hb) tetramers. These technologies were designed for the diagnostic investigation of hemoglobinopathies and therefore identify carrier states and other variants as by-products, resulting in large numbers of infants being followed up unnecessarily. The detection of carrier states as a by-product of NBS is of questionable clinical significance and has the potential to cause harms and anxieties as well as having significant cost implications. Tandem mass-spectrometry (MS/MS) has been used as an alternative technology for NBS as it is able to detect Hb peptides following the digestion of bloodspots with trypsin. Mutations generate peptides specific to the mutation present. The specificity of MS/MS means that haemoglobinopathies NBS can be limited to specified disorders, based upon screening policy and avoids unnecessary follow-up testing and referral of infants for genetic counselling. In addition, the use of MS/MS as a common platform within NBS laboratories allows the cost effective use of equipment/expertise that currently exist as the analysis for IMDs and haemoglobinopathies can be performed consecutively.

### O23. G6PD Newborn Screening

KhneisserIssamNewborn Screening Laboratory, Medical Genetic Unit, Saint Joseph University

Glucose-6-Phosphate Dehydrogenase deficiency is one of the most common X-linked disease. It is wide spread especially in regions related to Malaria exposure. It has been related to “favism crisis” since the ancient Greek empire. This hemolytic crisis triggered by oxidant exposure (like components of fresh fava beans) in G6PD deficiency cases need most of the time hospitalization. This food habit is very common in the MENA region. Kernicterus was highly reported in G6PD cases, birth stress on vulnerable red cells G6PD deficient increase of hemolytic crisis leading to neonatal jaundice.

Studies found early screening with adequate parents’ education prevent kernicterus, cerebral palsy and huge reduction of hemolytic crisis. In Lebanon, the risk of hemolytic crisis that need hospitalization due to food habit had been decreased from 78 per cent to less than 4 per cent. This end up with direct economic savings. Indirect benefit favor less emotional harm for families and reduce loss of productivity. All the above was used in many model of G6PD newborn screening cost benefit analysis.

### O24. Follow-up on a Positive Screening Result, Practical Issues

SkrinskaVictorDoha, Qatar

Positive screening results are considered a suspicious positive result that needs further follow-up which includes alerting parents, recall of the child, clinical monitoring of the child, sample collection, and confirmatory tests. In clearly symptomatic cases, treatment may be initiated before confirmatory results are available. However, in most cases treatment depends on confirmatory results. Recent advances have been made in reducing the time for differentiated diagnosis by reflexing initial positive screening results to 2nd tier biochemical or molecular tests applied to the initial dried blood spot (DBS). Application of 2nd tier biochemical tests have been shown to reduce false positive rates and help differential disorders. For example, an initial DBS with increased propionylcarnitine can be reflexed to provide levels of homocysteine, methylmalonic acid, and methylcitric acid combined with methionine to differentiate between multiple suspected disorders and false positive screens before they are reported as suspicious screens. More recently methods for routine extraction of DNA from DBS for rapid next generation sequencing enable the laboratory to confirm a suspicious positive result or identify a false positive result within a few days. These protocols have the potential to significantly reduce false positive rates and provide a rapid differential diagnosis to the attending physician.

### O25. Follow-up on a Positive Screening Result, Clinical Issues

SaadallahAmalNational Lab for Newborn Screening, King Faisal Specialist Hospital, Riyadh, Saudi Arabia

Newborn laboratory screening is effective only if the positive results are acted-upon through notifications and patient assessment. From a clinical perspective there is a need for examination to be followed by management then monitoring. 

Screening results are categorized as either high or low risk per Garg (2019). Primary communication of newborn screening (NBS) results with recommendations is made by NBS follow-up personnel to primary provider in USA (Percenti & Vickery (2019). In EU/Australia/New Zealand as per Chudleigh, Ren, Barben, and Southern, (2019) initial positive newborn screening (NBS) of cystic fibrosis (CF) is communicated to parents by a range of professionals including maternity ward/ midwife and CF center.

Categorizing clinical urgency include (1) Urgent—at immediate risk; (2) Urgent—not at immediate risk; (3) Important—not at immediate risk; and (4) Non-urgent (Chudleigh, et. al., 2019). The patient is contacted for clinical evaluation and additional laboratory testing for confirmation of screen-positive results (Garg, 2019). The ACMG (2019) ACT sheets and algorithms are for physicians to help them offer higher medical services, and to adherence to them. Example of guidance is to evaluate infant if poor feeding, lethargy, or hypotonia are present (ACMG, 2019). Moreover, to consult or refer to metabolic specialist to determine follow-up. Finally to undertake confirmatory testing in consultation with a metabolic specialist. Emergency care for infants with metabolic disorder or crises should be directed by a metabolic subspecialist in collaboration with emergency personnel and the family physician (Weismiller, 2017).

**References** 

ACMG American College of Medical Genetics and Genomics. Act Sheets and Lgorythms. 2019. Available online: https://www.acmg.net/ACMG/Medical-Genetics-Practice-Resources/ACT_Sheets_and_Algorithms.aspx (accessed on 18 February 2020).
  Chudleigh, J.; Ren, C.L; Barben, J.; Southern, K.W. International approaches for delivery of positive newborn bloodspot screening results for CF. *J. Cyst. Fibros.*
**2019**, *18*, 614–621. Available online: https://www.sciencedirect.com/science/article/pii/S1569199319300645#f0015 (accessed on 18 February 2020).
  Garg, U. Following up on Positive Newborn Screening Results. Ask the Expert. Clinical Laboratory News. July/August 2019. JUL.1.2019. Available online: https://www.aacc.org/publications/cln/articles/2019/julyaug/following-up-on-positive-newborn-screening-results (accessed on 18 February 2020).
  Percenti. L.; Vickery, G. Newborn Screening Follow-up. *North Carol. Med. J.*
**2019**, *80*, 37–41, doi:10.18043/ncm.80.1.37. Available online: http://www.ncmedicaljournal.com/content/80/1/37.full (accessed on 18 February 2020).
  Weismiller, D.G. Expanded Newborn Screening: Information and Resources for the Family Physician. *Am. Fam. Physician* 2017, 95, 703–709. Available online: https://www.aafp.org/afp/2017/0601/p703.html (accessed on 18 February 2020).

### O26. Ethics Considerations in Newborn Screening

OdaibAli AlNational Laboratory for Newborn Screening, King Faisal Specialist Hospital, Riyadh, Saudi Arabia

Newborn screening Program is one of the most effective public health Programs that’s lifesaving many newborns from an intellectual or physical disability, through early detection of a number of genetic conditions at their asymptomatic stage. Although those benefits of a newborn screening program outweigh harm and are beyond doubt, however, some ethical dilemmas do exist particularly with the advent in technology and expansion of conditions included in the program. Understanding some of the ethical consideration may help in guiding the development of an evidence- based decision that draws pace for more ethical legislations and policies. In this presentation, an overview of some of the worldwide ethical issues will be highlighted including the Saudi practice in the context of medical ethics fundamental rules and regulations.

### O27. The Importance of Cooperation between the Public and Private Sectors in Developing Countries in the Field of Newborn Screening

SahyounGeorgeMedLabs, Chief Scientific Officer, Amman, Jordan

The introduction of Newborn Screening within any society requires, at the very least, a high initial investment and highly technical staff. In developing countries, most government institutions are reluctant to take the necessary steps to introduce such a test, or even expand what is already in place to screen for a larger number of disorders.

This presentation is designed to stress the importance of cooperation between the private and public sectors in helping to provide all newborns with the most up-to-date, comprehensive and relevant test menu as per the prevalent disorders found within each society.

The presentation will give true real life examples of such success stories and how this has positively impacted on the services provided to newborns to help secure a more positive outcome for our future generations.

### O28. Quality Assurance and Quality Control Program for First Tier and Second Tier Testing

PetritisKostasCDC, Atlanta, USA

The Newborn Screening Quality Assurance Program (NSQAP) at the Centers for Disease Control and Prevention (CDC) is an accredited proficiency testing (PT) provider (ISO/IEC 17043:2010). NSQAP provides comprehensive dried blood spot (DBS) PT materials used by laboratories to achieve certification by regulatory bodies, and quality control materials (QC) for monitoring method performance. 

In 2019, CDC produced about 1,000,000 dried blood spots for 16 Proficiency Testing (PT) and 13 Quality Control (QC) programs that served the quality assurance needs of 685 laboratories from 85 countries participated. NSQAP collects PT data from participating laboratories and provides individual laboratory evaluations. 

In this presentation, after an introduction of the NSQAP program, emphasis will be given in the international PT and QC program availability, current data entry and reporting, 2017–2019 number of PT errors by country and main reasons for those errors. Forthcoming changes on the NSQAP data submission portal, the creation of lab specific statistics and new ways to visualize those data will be discussed. Finally, we will look at some preliminary results on harmonization activities for first and second-tier biochemical assays using the CDC QC materials.

### O29. Advances in Molecular Technology for the Follow up and Second Tier Testing

ChiniVasilikiHamad, Doha, Qatar

The advances in technology and automation, in the NBS laboratories, have enabled more efficient high-throughput screening, detection of more congenital conditions, as well as the incorporation of molecular methods to the newborn screening laboratory workflow.

In the Diagnostic Genomics Division (DGD), at Hamad Medical Corporation (HMC), newborns that are screen-positive or with an abnormal metabolic profile are rapidly followed-up with a molecular confirmation by Next-Generation Sequencing (NGS).

DNA is been extracted from NBS samples (both blood and Dried Blood Spots (DBS)) and the Whole Exome is been sequenced using the Ion Gene Studio S5 Prime machine (ThermoFischer Scientific). The analysis is performed by applying the in-house Newborn Screening Panel, which includes 122 genes, associated with 63 metabolic disorders and 18 additional NBS disorders.

35/40 WES results were in accordance with the initial clinical diagnosis based on the metabolic biochemical profile, while in five, no variation detected to support the suspected disorder based on the initial biochemical testing. Subsequent biochemical testing confirmed that these five cases were negative, confirming the NGS results.

From the newborn screening laboratory experience in Qatar, advances in molecular technology and the expansion of the recommended uniform newborn screening panel of diseases have led to earlier life-saving treatment and intervention.

### O30. Storage and Use of Residual Newborn Screening Specimens

KnowlesRachel LGreat Ormond Street Hospital for Children, London, UK

After newborn blood spot screening is complete, the UK programme stores residual samples for a minimum of 5 years to allow their further use in screening quality assurance, audit and development of laboratory methods, clinical investigations at the request of a child’s doctor and research. Consent taken from parents at the time of screening includes these purposes. In practice, residual blood samples are retained for different periods by screening laboratories with some holding residual blood spots for over 30 years. Access is carefully managed by a Research Advisory Committee which considers the public benefit of each research proposal, balancing this with the ethical implications and potential impacts on the screening programme. In recent years, requests for access to residual blood spots has risen with increasing recognition of the value of this resource for research, evaluation and audit aimed at improving health and screening programme performance.

### O31. Newborn Screening for Six Lysosomal Diseases: Current Status of a Pilot Study in Brazil

KubaskiFrancyneSouzaInêsAmorimTatianaPereiraDaniloTrometerJoeSouzaAlexandreRanieriEnzoPoloGiuliaHongXinyingGelbMichaelGiuglianiRobertoUFRGS/HCPA/INAGEMP, PPGBM, Porto Alegre, Brazil

Several lysosomal storage diseases (LSDs) already have specific therapies that, especially when introduced early, provide better outcomes. This study aims to evaluate the feasibility of newborn screening (NBS) for selected LSDs in Brazil, using tandem mass spectrometry (MS/MS). The study includes the screening of Gaucher, Fabry, Pompe, Krabbe, Niemann-Pick A/B, and Mucopolysaccharidosis (MPS) I. This is a prospective study in 20,000 unselected newborns from the state of Bahia, Brazil. The newborns with low enzyme activity will be further evaluated by biochemical and molecular genetics methods until the diagnostic status is clarified and they are referred for the appropriate management when indicated. The activity of the six lysosomal enzymes was analyzed with NeoLSD MS/MS kit (Perkin Elmer) on a Xevo TQ-S Micro (Waters). Validation of the method was conducted in dried blood spots provided by the supplier and on samples from newborns with these conditions. Instrument optimization was conducted to increase the signal and to decrease the in-source fragmentation. Initial cutoffs were established as a percentage of the median in nmoL/h/mL, as less than 0.97 (Gaucher), 1.18 (Fabry), 1.50 (Pompe), 0.30 (MPS I), 0.25 (Krabbe) and 0.60 (Niemann-Pick A/B). In a first run of 4,794 newborn samples, the number of samples with activities below the cutoff in the first assay was: 0 (Gaucher), 0 (Fabry), 3 (Pompe), 9 (MPS I), 9 (Krabbe) and 0 (Niemann-Pick A/B). All samples below the cutoff are being retested and if they continue to show enzyme activities below the cutoff, specific biomarker analyses and molecular genotyping will be performed in the same sample. The validation of this MS/MS method, initially in a pilot program with samples from 20,000 newborns, will provide important information about the feasibility of NBS for LSDs in Brazil.

### O32. High Incidence of Congenital Hypothyroidism in Pakistan Calls for Action: Need of a National Newborn Screening Program

MajidHafsaHabibAyshaJafriLenaHumayunKhadijahKhanNazishAga Khan University Hospital, Pathology and Laboratory Medicine, Karachi, Pakistan

Objective: Congenital Hypothyroidism (CH) is a serious disorder afflicting neonates which can drastically affect their mental health if left undiagnosed and untreated. In Pakistan, there were many studies regarding the prevalence of CH in different regions but the true incidence is not yet known. We sought to estimate the incidence of Congenital Hypothyroidism in the population of Pakistan.

Methods: A systematic review was conducted of studies reporting the incidence of CH in Pakistan from different areas. These studies included retrospective and prospective studies. Testing methods included Dried Blood Spot (DBS) or Serum testing with different diagnostic levels of Thyroid Stimulating Hormone (TSH). All sample sizes, age, and incidence results in the studies have been reported.

Results: Only 7 studies narrated the incidence of CH in our population from different areas of the country, based on screening in neonates from birth to 10 days of life using either Dried Blood Spot (DBS) or serum Thyroid Stimulating Hormone (TSH). Estimated incidence was observed to be from 1:250 to 1:1600, which is a higher incidence compared to the incidence reported by other developed countries (1:2000–1:4000).

Three studies performed DBS based TSH, screened a total of 7694 neonates and cumulative incidence was 1:769 using a cut off of 20 mIU/L. While remaining four studies screened neonates using different cutoffs of TSH, varying between 13–40 mIU/L.

Conclusion: This high incidence in Pakistan necessitates that a Newborn Screening program for CH should be implemented at a national level. It is a vital element of the healthcare system as it can lead to serious consequences including mental retardation.

### O33. A Comprehensive Evaluation of the Hellenic Newborn Screening: New People, New Technologies, New Possibilities

DimitriosPlatisGiorgosZolotasVasilikiGkioniDimitriosVasilakosPanagiotisGirginoudisInstitute of Child Health, Hellenic Newborn Screening Laboratory, Athens, Greece

Background**:** The Hellenic Neonatal Screening Program was initiated in 1974 and since then has been carried out by a single laboratory located at the Institute of Child Health (ICH) in Athens. The ICH tests the dried blood spot cards from all neonates born in maternity units in Greece (over 90,000 neonates/annum). Over the last 35 years, more than 3,700,000 neonates have been screened. Since the initiation of the Screening Program, metabolic disease experts, nutritionists, lab technicians, biologists and psychologists of the ICH, have been responsible for treatment initiation and clinical and laboratory follow-up of these patients.

Since, 2018 the Hellenic Neonatal Screening Program has been upgraded in multiple ways in an effort to improve services and provide more effective screening procedures and significant data analysis capabilities.

In preparation for the addition of Cystic Fibrosis in 2020 and efforts to introduce expanded screening we present an overview of the current situation of the Hellenic Newborn screening including several performance metrics.

Discussion**:** In this study we present our one-year review of our upgraded NBS Program, as well as, descriptive analysis of a one-year period for the screened disorders, PKU, Galactosemia, Congenital Hypothyroidism and G6PD Deficiency. 

### O34. PKU Patients Born before the Era of Population Newborn Screening: Is It Still Possible to Improve Their Life Quality?

GiżewskaMariaRomanowskaHannaKrzywińska-ZdebElżbietaPatalanMichałStrączekKamillaLeśniakAlicjaWalczakMieczysławPomeranian Medical University in Szczecin, Poland, Department of Paediatrics, Endocrinology, Diabetology, Metabolic Diseases and Ca, Szczecin, Poland

Phenylketonuria (PKU) is the most common inborn error of amino acids metabolism. If untreated hyperphenylalaninemia leads to severe brain damage and results in intellectual disability, speech difficulties, epilepsy, microcephaly and neurological impairment. Although in many countries neonatal population screening (NBS) was introduced in the mid-sixties of the last century, there are still regions where the procedure was started much later or is not performed at all. However, even in the countries with a long history of NBS there may exist numbers of patients with undiagnosed or untreated PKU. Many of them are dramatically impaired and often become the inmates of special institutions for disabled persons or live their life with the families being the source of many interfamilial problems. Their identification may create the opportunities to introduce the treatment with low-phenylalanine diet, which even started late, in some of them leads to a significant improvement in quality of life and facilitates every-day care. Patients become calmer with decrease in irritability, hyperactivity and aggressive behaviour. In some of them neurological symptoms decrease with reduction in the severity of epilepsy and improvement in the intellectual functioning. In the region of West Pomerania, Poland where PKU NBS was introduced in 1980, among 1016 adults with intellectual disabilities coming from 12 institutions for disable adults, 17 untreated or late-treated patients with PKU were diagnosed. In 8 cases treatment with a low-phenylalanine diet was introduced resulting in different degrees of improvement in 6 of them. However, it is hard to predict who will respond to dietary treatment-in many cases this type of intervention should be considered.

### O35. IT Infrastructure for Screening, Diagnosis and Long-Term Follow-up for Newborn Screening in Sweden

SörensenLeneZetterströmRolfKarolinska University Hospital, Centre for Inherited Metabolic Diseases, Stockholm, Sweden

The PKU test using dried blood spots (DBS) has been offered to newborns in Sweden since 1965. Today 25 diseases are included in the screening program and participation is over 99% of all newborns.

If an analysis in the screening gives a positive result, physicians at the PKU laboratory phone a pediatrician who recalls the child for diagnostic testing. If a metabolic disease is suspected the contact is made to one of four metabolic centers, for an endocrinological disease the contact is made to the child’s closest pediatric clinic and for an immunological disease the contact is made to one of three pediatric immunology clinics.

All children whose PKU sample yields a positive screening result are registered in a dedicated IT system at the PKU laboratory; “Larmsnurran”. The Swedish name roughly translates to “Recall carousel”, alluding to how positive results are reported out to pediatricians and back to the laboratory as being true or false.

After diagnosis, the entry is updated in “Larmsnurran” as a true or false positive. It is also updated with method of confirmation – biochemical or genetic. True positive entries for metabolic diseases are then exported to the Swedish national quality registry for inherited metabolic diseases (RMMS). Clinicians at the individual metabolic center can then access their patients’ basic data through RMMS and continue to register data for long-term follow up. Follow-up data generated at our laboratory is exported directly to RMMS.

This infrastructure facilitates a flow of information from the initial screening, through diagnosis, and finally long-term follow-up of patients. It also keeps track of true and false positives in a searchable database. All with minimal need for manual data entry.

### O36. The Use of 2nd Tier Blood-Spot Metabolites by Tandem Mass Spectrometry (MS/MS) to Reduce the False Positive Rate on Routine Newborn Screening for Inborn Errors of Metabolism (IEM)

RanieriEnzoMasEmilieGeraceRosemarieSA Pathology Women’s & Children’s Hospital, Biochemical Genetics, Genetics & Molecular Pathology, Adelaide, Australia

The use of 2nd tier blood-spot tests for a number of metabolites was added to the South Australian Neonatal Screening Programme to reduce the false positive recall rate associated with the primary measurement of amino acids, acylcarnitines and enzyme activities. These metabolites include the organic acids propionic, methylmalonic (MMS), succinic, 3-hydroxypropionic and orotic acids; the amino acids alloisoleucine & homocysteine; purines & pyrimidines, guanidinoacetate & creatine; lipids of 26:0-lysoPC, LysoGb1 & lysoGB3. These 2nd tier metabolites measured from the initial dried blood-spot collected at or near 48 hours of age enables to markedly enhance the sensitivity and specificity of the initial screening tests. The measurement of MMA is routinely used by many programmes to reduce the high false positive rate generated by propionyl-carnitine (C3) and to provide a specific marker for a group of IEM collectively known as methylmalonic acidemias. A non-derivatised blood-spot LC-MS/MS MMA method using a 5 × 100 mm Phenomenex C6-phenyl column with acetonitrile:water:formic acid at a flow rate of 150 µL/min directly into a QSight220 operated in negative ion mode. MMA was eluded from a 3 mm blood-spot and determined against (d3)-MMA using MRM pairs of 117.1/73.1 & 120.1/76.1 in a 5 minute isocratic LC run. Elevated MMA above 2.2 µmol/L whole blood, 99th centile required recall for plasma & urine MMA and B12 determination. This method enables the detection of neonates with B12 deficiency, MMA and disorders of Cobalamin, having identified cases of cblA & cblD deficiencies and neonates with significant B12 deficiencies and who have been treated. The determination of other 2nd tier metabolites such as 3-hydroxy-propionic acid, homocysteine, 26:0-LysoPC, LysoGb1 & LysoGb3 will be presented to show the value of the inclusion of these markers in reducing the false positive rate from the initial dried blood-spot for the neonatal screening of these selected disorders.

### O37a. Routine Newborn Screening of Newborns—Why Can’t ISNS & other International Organisations Make it Mandatory in Asia?

KumarKishoreCloudnine, Dept. of Neonatology, Bangalore, India

Study Objectives: Routine newborn screening programme for various disorders was started mandatorily in our group of hospitals 11 years ago for 2 reasons: World over there is enough evidence of Newborn Screening Saving Lives (NSSL) and to see if it’s applicable to Indian subcontinent?

Methods: Screening for various disorders including congenital hypothyroidism (CHT), Glucose-6-Phosphatase Dehydrogenase Deficiency (G6PD), Congenital Adrenal Hyperplasia (CAH), Galactosaemia (GLT), Biotinidase deficiency (BD), metabolic disorders along with screening for disorders like Developmental Dysplasia of Hips (DDH), Retinopathy of Prematurity (ROP), Newborn Hearing Screening (NHS), Screening for Critical Cyanotic Congenital Heart Disease (CCCHD) was performed on all babies born from various hospitals in the group in South India who consented to be part of this between 19th January 2007 and 19th January 2018.

Results: In the study period, a total of 69,426 babies were screened. There were 86 cases of CHT (incidence of 1:807), 1735 cases of G6PD (incidence of nearly 2.5%), 27 cases of CAH (incidence of 1:2500), 2 cases of Galactosaemia (incidence of 1: 34,713), 1 case of Biotinidase deficiency (incidence of 1:69,426), many metabolic disorders—almost 245 cases—0.35% incidence-predominant among them being Urea Cycle Defects (UCD—10 cases of Citrullinaemia-incidence of 1:6942), 4 cases of deafness (amounting for 1:17,356 cases), 13 cases of DDH (incidence of 1:5340) and 24 cases of CCCHD (incidence of 1:2900), 4 cases of ROP (incidence of 1:17,350)—suggesting that there is enough proof in the pudding to enforce mandatory screening in all member countries.

Conclusions:

1. ISNS has a responsibility for all members and countries with its knowledge base.
2. Why can’t ISNS issue a statement asking member countries—which can save lives and help decrease morbidity and mortality (and reduce IMR in member countries).

### O37b. Newborn Screening: Why Is Asia Lagging behind & What Can They Do?

KumarKishoreCloudnine, Dept. of Neonatology, Bangalore, India

Newborn screening is the most important preventive public health programme of the 21st century. India and many countries in Asia are yet to start any publicly funded programme despite this having been established practice in many countries for over 50 years.

Asia is having one of the highest Infant and Neonatal Mortality Rates (IMR & NMR) in the world. Without newborn screening it is highly unlikely that we will ever achieve single digit NMR.

Most people in India & Asia do not know newborn screening, including many medical professionals. Once a country decides to implement screening—then what to screen for? This depends upon several factors.

In India, currently we have enough data to suggest that we can implement screening for 4 major diseases, which are either making our babies die or disabled for no fault of theirs nor their families.

The incidence of these 4 diseases is much more than the rest of the world and the cost of the screening for these 4 diseases should not be more than Rupees 500 (less than $USD8—but most people try and make a huge profit when they offer these 4 diseases screening—which leads to it being rejected by many people for the cost and lack of awareness.

Though systematic neonatal screening for congenital hypothyroidism was introduced in the early 1970s in many countries; in India an estimated 10,000 babies are born with congenital hypothyroidism every year, yet there is no screening programme for this. We seem to have started hypothyroidism screening in most parts of India now. With this hopefully our scope of newborn screening will change.

There is enough data present in India to start the screening process for 4 diseases and probably improvise adding few more diseases later–just like Philippines have started. Asia should follow the rest of the world in trying and saving lives by collecting their own evidence and incidence of diseases & implement newborn screening accordingly—because NEWBORN SCREENING SAVES LIVES.

## Poster Presentations

### P1. Combined Effect of Gestational Age and Birth Weight on Metabolites Related to Inherited Metabolic Diseases in Neonates

WangLingYiFangChengdu Newgenegle Clinical Laboratory, Clinical department, Chengdu, China, People’s Republic

Objective To study the combined effect of gestational age and birth weight on metabolites related to inherited metabolic diseases (IMD). 

Methods A total of 3381 samples ruled out of IMD by follow-up were randomly selected from 38931 newborns during 2014–2016. These neonates were categorized into seven groups according to their gestational age and birth weight: extremely preterm appropriate- for-gestational age (e-AGA) group (*n* = 12), preterm small-for-gestational age (p-SGA) group (*n* = 18), preterm AGA group (p-AGA, *n* = 219), preterm large-for-gestational age (LGA) group (p-LGA, *n* = 18), full-term SGA group (f-SGA, *n* = 206), full-term AGA group (f-AGA, *n* = 2677), and full-term LGA group (f-LGA, *n* = 231). Levels of 17 key IMD-related metabolic indices in dried blood spots were measured using tandem mass spectrometry. Spearman’s correlation analysis was used to investigate the relationships between 17 indices and their influencing factors, while covariance analysis was used to compare the metabolic indices between these groups. 

Results After adjusting the influencing factors such as physiological and pathological status, compared with f-AGA, e-AGA, p-SGA, and p-AGA groups had significantly reduced levels of leucine\isoleucine\hydroxyproline and valine (*p* < 0.05); p-AGA group had a significantly decreased ornithine level (*p* < 0.05); e-AGA and p-AGA groups had a significantly reduced proline level (*p* < 0.05). Besides, the methionine, free carnitine, acetylcarnitine, and propionylcarnitine level in the p-AGA and p-SGA groups had significantly increased (*p* < 0.05). The phenylalanine and tyrosine also significantly increased in p-AGA groups (*p* < 0.05).

Conclusions Low gestational age and low birth weight may result in abnormal results in IMD screening. Therefore, gestational age and birth weight should be considered to comprehensively judge the abnormal results in IMD screening.

### P2. Should Genetic Markers be Nominated for the New Expanded NBS Panel?

ZeglamAdelTarhooniSamiraTripoli University Hospital, Pediatrics, Tripoli, Libya

Inborn errors of metabolism are alone rare but jointly common diseases. A large proportion of these diseases are treatable such that neurological damage can either be reversed or prevented. Several reports had previously shown DNA could easily be extracted from the Guthrie spot and analyzed successfully for genetic markers. A surprisingly large number of IEMs can present with CP symptoms, as ‘CP mimics’, and can be identified with minimally invasive testing. 

Case Report: To report new variants of SLC39A14 gene mutations in a 3 years old female and 5 years old male and heterozygous variant in SPR gene mutation in 8 years old female all of whom presented with stiff muscles and dystonia. To the best of our knowledge these variants have not been described in the literature so far. 

Conclusion: This inborn error of manganese metabolism has only recently been identified. Mutations in the SLC39A14 gene have been linked to autosomal recessive hypermanganesemia with dystonia type2.Whole exome sequencing (WES) identified the homozygous missense variant {c.1136C>T p. (Pro379Leu)} and {c.77dup C p. (Glu27)} in exon 7 of the SLC39A14 gene. Mutations in SPR gene have linked to autosomal recessive/dominant dopa responsive dystonia, WES identified the heterozygous variant {c.207C.G, p. (Asp69Glu)} in SPR gene. Hypermanganesemia and Sepiapterin Reductase deficiency (SRD) should be considered in any child that presents with dystonia. 

Discussion: Clinician attentiveness of treatable CP mimics is important for appropriate screening, diagnosis and early intervention. Due to state of art advances and developments in screening technology in the last decade, there has been a lot of changes in the way that we diagnose children with inborn metabolic diseases as more biomarkers become identified. In Libya few families who are affected with such disorders undergo molecular diagnosis.

### P3. Newborn Screening for G6PD Deficiency and the Mutational Spectrum in Vietnam

LuyenQuoc HaiBionet Vietnam, Newborn screening center, Ha Noi, 10000, Vietnam

Vietnam belongs to a group of countries in Southeast Asia where the potential risk of glucose-6-phosphate dehydrogenase (G6PD) deficiency is associated with a high prevalence of malaria. Study and screening of G6PD deficiency in the Vietnamese population is still scant and fragmented. We present the first nationwide screening study to examine the rate and spectrum of mutations causing G6PD deficiency in Vietnam.

### P4. 14 Years Experience in Expanding Newborn Screening Using MSMS and Confirmation Positive Results in KFSHRC, Saudi Arabia

AlamoodiMohamed SKFSHRC, Genetic, Riyadh, Saudi Arabia

Newborn Screening is a public health program of screening in infants shortly after birth for a list of condition that are treatable, but not clinically evident in the newborn period, In 1994 metabolic screening lab in KFSHRC introduced expand newborn screening for amino acid disorders, fatty acid oxidation and organic acidemia. In 2005 the Saudi newborn screening program start.

The talk will include Newborn Screening in Saudi Arabia, sampling (sample from baby, laboratory process, injection to the MSMS and interpretation for normal and positive and finally MSMS operation. 

### P5. Development of a HILIC-LC-MS Analytical Method to Screen Pyridoxine Dependent Epilepsy from Dried Blood Spot

MoorkothSudheerMathewElizabeth MaryLewisLeslieRaoPragnaManipal College of Pharmaceutical Sciences, Pharmaceutical Quality Assurance, Manipal, India

Background: Pyridoxine dependent epilepsy (PDE) is a metabolic disorder where the affected newborns have prolonged seizures within the first month of life. These seizures will not respond to anticonvulsant therapy. However, if identified earlier they can be treated prophylactically with pyridoxine and a severe brain damage can be prevented. Pipecolic acid (PA), Alpha amino adipic semialdehyde (AASA) and Piperidine-6-carboxylic acid (P6C) are known to be the markers for this condition. Development of an analytical method for the simultaneous quantification of these markers from dried blood spot (DBS) is reported here.

Methods: Dried blood spots were prepared from blood samples of neonates of 3–5 days of age born at Kasturba Hospital, Manipal after obtaining ethical clearance (MUEC/010/2017 dtd 08/05/2017). The chromatographic conditions and sample extraction procedure for DBS were optimized by varying different experimental parameters. The optimized method was validated as per the guidelines.

Results: The separation of biomarkers was optimized on the iHILIC column with the following conditions. Mobile phase (acetonitrile: formic acid buffer, 80:20 v/v ratio), flow rate 0.5 mL/min, Injection volume 10 µL, and run time 3 min. The retention time of PA, AASA/P6C and d9-PA were 1.79, 2.59 and 2.20 min respectively. MS conditions were optimized for spray voltage, vaporizer temperature, sheath gas flow and for collision energy. For extraction of sample, the spot size was optimized to 3.2 mm DBS. Extraction was affected with 100µL of methanol with vortexing for 30 min at room temperature. The LOQ of the method was found to be 10 ng/mL and 50 ng/mL respectively for AASA/6PC and PA respectively.

Discussion: The iHILIC column facilitated the retention of PA and AASA without derivatization and has a low run time of 3 min. The validation of the method demonstrated a good linearity, accuracy and precision. The method can be applied for newborn screening of PDE.

### P6. An Innovative SMA Screening Method Directly from Dried Blood Spots

VandermeulenCharlotteGiltayAxelDetemmermanLiselotLaCAR MDx, Molecular diagnostic, 4102 Liège, Belgium

Spinal muscular atrophy (SMA) is a hereditary neurodegenerative disease with an average birth incidence of 8 per 100.000 persons. SMA is categorised in 4 types depending on clinical features. However, most SMA cases belong to the most grievous category and symptoms often lead to death. Two highly similar genes, SMN1 and SMN2 encoding the SMN protein, are associated with the disease. SMN1 is crucial for disease onset as in 95% of SMA patients the SMN1 gene is truncated and/or is converted to SMN2. SMN1 conversion to its homologous gene is caused by a silent mutation in the exon 7 of SMN1 (c.840C>T), which leads to exclusion of exon 7 and the expression of a less stable SMN protein.

New therapeutic drugs have shown promising results when treatment is started early in infancy, SMN1 newborn screening is essential to rapidly detect and treat SMA. We have developed a screening technique for the detection of SMN1 exon 7 presence and of the c.840C>T variant directly from dried blood spots, without the need for DNA extraction. 

SMN1 disease-related variants are detected using LAMP PCR. After amplification, presence of SMN1 exon 7 is determined during an annealing step. Patients presenting an annealing peak for SMN1 are positive for at least one copy of SMN1 gene. When a peak for the reference gene is detected, while no peak is present for SMN1, the patient does not possess a wild type copy of SMN1. In addition, patients positive for a copy of SMN1 can be analysed for the 840 C<T point mutation by melting curve analysis. The analysis establishes if there is at least one SMN1 allele that is wild type and therefore fully functional.

Our technique allows for a rapid detection of SMN1 truncation and/or conversion for identification of SMA. Clinical validation is ongoing at the Dept of Human Genetics, University of Liège (Belgium) in collaboration with Pr. Vincent Bours and Dr. François Boemer. We plan to finish CE-marking beginning 2020 and to implement an automated method for newborn screening as a first molecular diagnosis method

### P7. Development and Validation of a Spatially Multiplexed Digital Microfluidics Platform to Screen for Biotinidase Deficiency and Galactosemia

KalelkarSandeepBrannenCandiceWashburnJonPatelHariUllalAnirudhChopraSallyBillingsDavidMunDanielBaebies, Newborn Screening, Durham, United States

Newborn screening (NBS) for the disorders of Biotinidase deficiency and galactosemia has been performed in the United States for several years and there is an increasing level of global interest to implement NBS for these disorders. We have expanded the capabilities of our digital microfluidic (DMF) cartridge and platform to multiplex fluorimetric assays for the measurement of galactose-1-phosphate uridyltransferase (GALT) and biotinidase (BIOT). The assays are run on the same platform that is FDA cleared to screen for the four lysosomal storage disorders Pompe, Fabry, Gaucher and MPS 1. The BIOT assay generates a fluorimetric signal using 4-methylumbilliferone, while the GALT assay is based on the NADPH fluorescent readout from a 3-step enzyme cascade. There is a strong need to standardize the assays used to screen for BIOT and GALT as public health laboratories currently run one of five different assays that screen for galactosemia and several BIOT screens use “home-brew” assays. Some of these assays are even qualitative and non-quantitative. This makes it difficult to directly compare the performance and results from different laboratories. We have developed fully quantitative assays that monitor the enzymatic activity associated with BIOT and GALT. In order to demonstrate the performance of these two assays, we have analytically validated them according to CLSI guidelines. This validation includes single, multi-day, and multi-instrument precision, interference analyses, method comparisons to currently performed assays, carryover, linearity, and analyses for limits of detection and quantitation.

### P8. Determination of Multplex Enzyme Assay Activities in DBS Samples Using NeoLSD KIT and LC-MS/MS

ObeidatNajahOkourEsraaJordan university of science and technology, Princess Haya Biotechnology Center, Irbid-Jordan, Jordan

Background: The application of tandem mass spectrometry gaining territories in many fields, lysosomal storage disorders (LSD) compounds related to enzymes activities had been included among the multiplexes that can be analyzed on MSMS. The number of LSD related multiplexes can reach up to 19 metabolites. Since 2009, Princess Haya Research center in Irdid, North of Jordan, started to use this application in his diagnosis facilities for six-plex. We will present our results during the last decade and the recent switch from homebrew prepared CDC free solutions to the use of the NeoLSD kit.

Methods: Dried blood samples were collected on Whatmann 903 from patient referrals based on clinical assumptions, CDC LSD free materials and method had been used from 2009 till 2018 on API3200. In 2019, we did switch to use The NeoLSD kit. Both methods are characterized by an overnight incubation and Liquid-Liquid extraction.

CDC NSQAP was used as our external quality control program.

Results: Among 1000 DBS samples analyzed for diagnostic of LSDs, we found 86 confirmed positive cases, 4 Krabbe, 4 Fabry, 33 Pompe, 25 gaucher, 8 MPS I and 12 Niemann-pick A/B.

Discussion and Conclusion: The high incidence found is in favor to add Newborn screening for lysosomal storage disorders (LSD). A Cost-effective study could be an additional tool for decision makers to add LSD screening to our National Panel. The research of the etiology and the genetic founding of this high incidence is needed to highlight more about consanguinity and isolated tribes marriage tradition, etc.

### P9. Quantitation of Glycosaminoglycans In Amniotic Fluid by Liquid Chromatography Tandem Mass Spectrometry: A Potential Tool for the Rapid Prenatal Identification of MPS in Pregnancies at Risk

KubaskiFrancyneKesslerRejane G.MachadoAndryele Z.MoraesInamara S.MedeirosFernandaBenderFernandaBurinMaira G.Michelin-TirelliKristianeHorovitzDafne D.G.BarthAneliseMasonRobert W.TomatsuShunjiGiuglianiRobertoUFRGS/HCPA/INGAMEP, PPGBM, Porto Alegre, Brazil

Mucopolysaccharidoses are multisystemic life-threatening lysosomal storage disorders caused by the deficiency of lysosomal enzymes that lead to the accumulation of glycosaminoglycans (GAGs). Due to family history, prenatal diagnosis in pregnancies at risk is occasionally performed. We have previously demonstrated that MPS VII fetus has a very high accumulation of GAGs in amniotic fluid (AF). To elucidate if MPS I and MPS II fetus also have prenatal accumulation of GAGs detectable by liquid chromatography tandem mass spectrometry (LC/MS/MS) in AF, we have analyzed samples of 2 MPS fetuses (1 MPS I and 1 MPS II) and compared with 3 age-matched controls. MPS diagnosis in the MPS cases was confirmed by enzyme assays in amniocytes (alfa-iduronidase for MPS I, and iduronate-2-sulfatase for MPS II). Disaccharides of heparan sulfate (HS-NS and HS-0S) and dermatan sulfate (DS) were obtained from the AF supernatant immediately after collection by digestion with heparitinase and chondroitinase B and were quantified by LC/MS/MS. Values were normalized by creatinine and expressed and ng/mg of creatinine. Both MPS fetuses had elevated levels of GAGs in AF, with at least 2 fold increases for DS, and more than 8 fold increases of HS-NS and HS-0S. The quantification of GAGs in AF in at-risk pregnancies, which could provide results a few hours post AF collection, may be useful to quickly provide useful information regarding prenatal MPS diagnosis, well before results of enzyme assay (which usually requires the cultivation of amniotic fluid cells that may take 2 to 3 weeks to grow) or molecular genetic analyses are provided.

### P10. Biochemical and Molecular Characterization of CTNS Mutations in Tunisian Patients with Cystinosis

GafsiRouaidaChkiouaLatifaDandanaAzzaBahiaWaelBoudabousHelaBouzaabiaMaroua NouiriBrahimMaissaTebibNejiLimemKhelifaFerchichiSalimaUniversity of Monastir, Faculty of pharmacy, 5000 Monastir, Tunisia

Nephropathic cystinosis (NC) is an autosomal recessive disorder characterized by defective transport of cystine across the lysosomal membrane and resulting in renal, ophthalmic, and other organ abnormalities. Mutations in the CTNS gene cause a deficiency of the transport protein, cystinosin. This study was performed to investigate mutations of the CTNS gene in three Tunisian families with NC.

Polymerase chain reaction (PCR), ARMS multiplex PCR and direct sequencing were performed for molecular characterization of the CTNS gene in 3 unrelated Tunisian patients and their parents. Based on family history, prenatal diagnosis (PND) was performed in fetal DNA isolated from chorionic villi obtained at 10–12 weeks of gestation.

None of the patients showed the most common 57-kb deletion in heterozygous or homozygous status. One patient was homozygous for the previously reported mutation c.1515G > A (p.G308R). One patient presented the novel gross deletion of 20,327 bp. One was homozygote for the previously reported mutation c.771_793del (p.Gly258Serfs*30). In addition, eight polymorphisms were identified in the 3 patients and their parents. The prenatal diagnosis in one family showed that the fetus DNA was heterozygous for the c.771_793del (p.Gly258Serfs*30) mutation.

This study expands the mutational and population spectrum of NC, representing the first molecular diagnosis of NC in Tunisian population. The mutation screening of the CTNS gene was used for prenatal diagnosis to prevent and/or limit this inheritable disease in our country where the families are particularly large and have a high rate of consanguinity.

**Keywords:** Nephropathic cystinosis, CTNS, Mutations, Polymorphisms, Tunisian families, Prenatal diagnosis

### P11. Profile of Mucopolysaccharidoses Diagnosed at the Biochemistry Laboratory in Farhat Hached Hospital–Sousse Tunisia

BahiaWaelDandanaAzzaChamekhZinaBoudabousHelaBouzaabiaMaroua NouiriBrahimMaissaTebibNejiLimemKhelifaFerchichiSalimaFarhat HACHED Hospital, Laboratory of Clinical Biochemistry, Sousse, Tunisia

Introduction: The mucopolysaccharidoses (MPSs) are a group of rare genetic disorders of glycosaminoglycan (GAG) catabolism). Each MPS disorder is caused by a deficiency in the activity of a single, specific lysosomal enzyme required for GAG degradation.

Objective: The aim of this work is to describe the epidemiological characteristics of MPSs diagnosed in our laboratory, going from 2010 to the year 2018.

Results: In our study, we detected that the number of applications sent for the diagnostic of MPS are decreased: 91 requests in 2010 against 58 requests in 2018. Then, for 648 applications, we detected for MPS 47 (7.2%) positive cases. 20 cases (42.5%) presented Sanfilippo Syndrome (MPS III), 8 cases (17%) had Hurler syndrome (MPS I), 4 cases (8.5%) with Morquito syndrome (MPS IV). For MPS II, VI and VII, 2 cases were noted for each. The enzymatic activity to differentiate type I from type II was not done for the rest of the cases (19%).

Conclusion: Lysosomal storage disorder, and especially MPSs, constitutes, in Tunisia, a public’s health problem. We identified 47 children with MPSs from 2010 to 2018. To reduce the incidence of these diseases the only alternative is the prenatal diagnosis carried out in families at-risk.

### P12. The Metachromatic Leucodystrophy: Experience of Laboratory of Clinical Biochemistry In Farhat HACHED Hospital

BahiaWaelDandanaAzzaChamekhZinaBoudabousHelaBouzaabiaMaroua NouiriBrahimMaissaTebibNejiLimemKhelifaFerchichiSalimaFarhat HACHED Hospital, Laboratory of Clinical Biochemistry, Sousse, Tunisia

Introduction: Metachromatic leukodystrophy is a lysosomal storage disease caused by a deficiency in arylsulfatase A. Clinically, three phenotypes were distinguished: Late infantile, juvenile and adult form. These phenotypes vary according to the absence or presence of neurological manifestations and their progression degree.

Our purpose was to demonstrate the frequency of requests for the determination of arylsulfatase A activity in our laboratory.

Methods: We conducted a retrospective study for 6 years (2013–2018) including all requests for the arylsulfatase A assay sent to our laboratory. The measurement of arylsulfatase A enzyme activity was performed in leucocytes by a colorimetric method using a synthetic substrat: paranitrocathechol-sulfate.

Results: During our study, 139 requests were sent to our laboratory: 8 requests in 2013, 15 requests in 2015, 43 requests in 2015, 31 requests in 2016, 10 requests in 2017 and 32 requests in 2018. We detected 11 patients with a deficit of arylsulfatase A activity (7.9%) in our study population during these 6 years: four cases in 2018, two cases in 2017, four cases in 2016 and one case in 2014.

We found four cases having the infantile form (≤2 years), five patients having the juvenile form, and two cases having the adult form.

The average of enzymatic activity level of the arylsulfatase A at 37 °C was 3.68 μkat/kg of protein with extremes from 1.25 μkat/kg to 10 μkat/kg of protein (NV: 12–35 μkat/kg of protein). At 0 °C, the average of enzymatic activity level was 0.33 μkat/kg protein with extremes from 0 μkat/kg to 1.8 μkat/kg protein (NV: 3–9 μkat/kg protein).

Conclusion: Metachromatic leukodystrophy is a very disabling hereditary disease. Unfortunately, the specific treatment of this disease is not always available in Tunisia, therefore, required to the prenatal diagnosis and genetic counseling.

### P13. Clinical, Biological and Molecular Profiles of Fabry Disease in Tunisian Families: A Case Report

ChkiouaLatifaSaheliChaimaBoudabousHelaJaafouraLamiaFerchichiSalimaTebibNejiLaradiSandrineUniversity of Monastir, Faculty of pharmacy, 5000 Monastir, Tunisia

Background: Fabry disease (FD) is an X-linked disorder caused by the deficiency of the lysosomal enzyme alpha-galactosidase A (α-GAL). The present study is aimed at performing the molecular characterization of Tunisian families with Fabry disease.

Patients and methods: The clinical diagnosis was accomplished by demonstration of the deficient α-GAL activity in leucocytes of two unrelated patients and automated sequencing of the seven exons of the GLA gene.

Results: For mutation detection, each of the 7 exons and adjacent intron–exon junctions of the GLA gene were sequenced after PCR-amplification from genomic DNA. In the present study, two mutations were identified in the two families whose child respectively presents the classical subtypes of FD: c.803+2T>C (IVS5+2T>C) in intron 5 and p.L394P (c.1181T>C) in exon 7.

The first previously identified mutation was a C to T transition in the conserved 5’ donor splice site of intron 5 (ATGgtaaaa>ATGgcaaaa): designated IVS5+2T>C). The nucleotide variation of the second patient was a missense mutation p.L394P, resulting in the substitution of thymine by cytosine at 1181 position of genomic DNA. These mutations were not found in the 100 allele’s controls.

In addition, four novel variations c.-291T>C, IVS4+22 (c.644C>G), IVS6-24delCA (c.1000-24delCA) and IVS3-12C>T and three previously reported polymorphisms, rs3027584G>A, rs782116078C>T and rs782506652 were identified in studied families.

Conclusions: These molecular findings allow improving genetic counseling, as well as accurate carrier detection including female heterozygote detection, prenatal diagnosis, and counseling for Fabry disease in Tunisia.

### P14. Gaucher Disease in a Tunisian Family

DandanaAzzaBahiaWaelChamekhZinaBoudabousHelaBrahimMaissaTebibNejiLimemKhelifaFerchichiSalimaFarhat HACHED Hospital, Laboratory of Clinical Biochemistry, Sousse, Tunisia

Introduction: Gaucher disease is an inborn, autosomal recessive error of the metabolism which belongs to the group of lysosomal storage disorders.

Objective: This work reports on the diagnosis of Gaucher disease in several members of the same family from Tunisia.

Methods: This was a descriptive case study about the biochemical and molecular diagnosis of Gaucher disease.

Results: The diagnosis of these patients was performed by measuring the levels of glucocerebrosidase and confirmed by genotyping. All patients suffering from Gaucher disease had low glucocerebrosidase activity (0, 1 to 0, 5 µkat/gr proteins (reference value: 3’9 µkat/gr proteins). Hepatosplenomegaly was the most common clinical manifestation (100%) and osteopenia was seen in 80% of the cases. Regarding hematological manifestations, anemia and leukopenia were found in 40% of patients at diagnosis. Our results showed that two members (Father and uncle) of this family were homozygote for the N370S ((c.1226 A>G) mutation, two members (children) were heterozygotes for this mutation. the mutation is absent in the mother.

Discussion: The prevalence of the N370S mutation in Tunisian GD patients is one of the highest in Arab populations. In such populations, the number of mutations giving rise to GD is small, which facilitates a rapid genetic diagnosis.

Conclusion: Gaucher’s disease is not exceptional in Tunisia. Type 1 is the most common type. In these cases, the treatment as the family with Gaucher disease showed possible and necessary.

### P15. N370S (c.1226 A>G) Mutation among Tunisian Patients with Gaucher Disease

DandanaAzzaBahiaWaelChamekhZinaBoudabousHelaBouzaabiaMaroua NouiriBrahimMaissaTebibNejiLimemKhelifaFerchichiSalimaFarhat HACHED Hospital, Laboratory of Clinical Biochemistry, Sousse, Tunisia

Gaucher Disease (GD) is a most common type among lysosomal storage disorders. It is an autosomal recessive disease, caused by deficiency of an enzyme ß-glucocerebrosidase (GBA).

Methods: The GBA activity was performed in leucocytes using the fluorescent substrate, 4-methylumbelliferone ß-glucoside. The amount of 4-methyl–Umbeliferone (4–UM) was quantified using a fluoremetric method (excitation length: 340 nm, emission: 495 nm). All patients were screened for four mutations in the GBA gene (N370S, 84dupG, IVS 2 (+1) G>A and L444P). All mutations were analyzed by polymorphism chain reaction (PCR) amplification and restriction enzyme digestion.

Results: 256 patients enrolled in our clinical laboratory of Biochemistry in Tunisia, were diagnosed for GD.

Among all patients, only 15 presented Gaucher disease (decreased activity of GBA, splenomegaly in 12 (80%), hepatomegaly in 8 patients (53%) and thrombocytopenia in 4 (26%). Skeletal manifestations were detected in 4 cases (30%). Of the 15 individuals screened, 9 were found to be homozygotes for the N370S ((c.1226 A>G) mutation and two were found to be heterozygous for it. Two patients with the acute neuronopathic type who presented with hydrops fetalis harbored the L444P mutation.

For the fifteen patients, L444P was found only in 20% of alleles, N370S in 60% of alleles, and 84dupG was detected in two alleles (3, 57%). IVS 2 (+1) G>A has not been detected in our study population.

Discussion: The N370S mutation results in a catalytically deficient enzyme with normal or near normal levels. The N370S mutation influences the flexibility of the loop 1 region resulting in reduced catalytic activity.

According to several studies, Tunisian patients show a profile characteristic of the Arab and the south Mediterranean populations, with the N370S mutation being the most prevalent, succeeded by the L444P, the D409H and the RecNCi I mutations.

Conclusion The prevalence of the N370S mutation in Tunisian GD patients is one of the highest in Arab populations.

